# Underpinning the molecular programming attributing heat stress associated thermotolerance in tea (*Camellia sinensis* (L.) O. Kuntze)

**DOI:** 10.1038/s41438-021-00532-z

**Published:** 2021-05-01

**Authors:** Romit Seth, Tony Kipkoech Maritim, Rajni Parmar, Ram Kumar Sharma

**Affiliations:** 1 Biotechnology Department, CSIR-Institute of Himalayan Bioresource Technology (CSIR-IHBT), Palampur, Himachal Pradesh 176061 India; 2 Academy of Scientific and Innovative Research (AcSIR), CSIR-HRDC Campus, Ghaziabad, Uttar Pradesh 201002 India; 3 Tea breeding and genetic improvement division, KALRO—Tea Research Institute, Box 820, 20200 Kericho, Kenya

**Keywords:** Heat, Transcriptomics, Abiotic, Drought

## Abstract

The most daunting issue of global climate change is the deleterious impact of extreme temperatures on tea productivity and quality, which has resulted in a quest among researchers and growers. The current study aims to unravel molecular programming underpinning thermotolerance by characterizing heat tolerance and sensitivity response in 20 tea cultivars. The significantly higher negative influence of heat stress was recorded in a sensitive cultivar with reduced water retention (47%), chlorophyll content (33.79%), oxidation potential (32.48%), and increase in membrane damage (76.4%). Transcriptional profiling of most tolerant and sensitive cultivars identified 78 differentially expressed unigenes with chaperon domains, including low and high molecular weight heat shock protein (HSP) and heat shock transcription factors (HSFs) involved in heat shock response (HSR). Further, predicted transcriptional interactome network revealed their key role in thermotolerance *via* well-co-ordinated transcriptional regulation of aquaporins, starch metabolism, chlorophyll biosynthesis, calcium, and ethylene mediated plant signaling system. The study identified the key role of HSPs (*Cs*HSP90) in regulating HSR in tea, wherein, structure-based molecular docking revealed the inhibitory role of geldanamycin (GDA) on *Cs*HSP90 by blocking ATP binding site at N-terminal domain of predicted structure. Subsequently, GDA mediated leaf disc inhibitor assay further affirmed enhanced HSR with higher expression of *Cs*HSP17.6, *Cs*HSP70, HSP101, and *Cs*HSFA2 genes in tea. Through the current study, efforts were made to extrapolate a deeper understanding of chaperons mediated regulation of HSR attributing thermotolerance in tea.

## Introduction

Tea (*Camellia sinensis* (L.) O. Kuntze), a highly heterogeneous self-incompatible tree species of family theaceae is widely cultivated in the tropical and sub-tropical regions of the world^1^. The leaves of the tea plant were globally served as a hot/cold rejuvenating brew, which is rich in taste, flavor, aroma, and medicinal properties due to enriched secondary metabolites^2^. Considering the high global demand, tea production is a major source of revenue in South-east Asian and African countries^3^. Nevertheless, climate-driven abiotic and biotic factors resulted in a significant reduction in the quality and global production of tea^4–6^. According to the intergovernmental panel on climate change (IPCC), global warming triggered by the greenhouse effect is one of the major threats to most of the tea-growing regions^7^. During the last five decades, the global temperature has recorded a significant rise from 0.19 °C/decade to 0.25 °C/decade, which is even faster than the mean annual temperature^8^. Current projections indicate that constantly raising ambient temperature may restrict the spatial distribution of tea cultivation by inhibiting its growth, yield, and quality in various tea-growing regions^9,10^. However, a sustainable increase in tea production requires intensified efforts through multi-targeted approaches to develop climate-resilient tea cultivars with improved yield and quality.

The heat stress is known to severely affect the membrane fluidity, which causes malfunctioning of intracellular organelles (chloroplast and mitochondria) due to increased cytosolic calcium and reactive oxygen species (ROS)^11^. The increased ROS seldomly leads to over-reduction of electron transport chain (ETC) by activating photorespiration, Mehler’s peroxidase activity, and also decreases photosynthesis efficiency in C3 plants^12–14^. Nevertheless, various plant species respond to thermal stress (TS) by activating heat shock response (HSR) with the help of molecular chaperons for intracellular protein stabilization and cellular homeostasis^11^. These chaperones (heat shock proteins, HSPs) were regulated *via* activation of heat shock transcription factors (HSFs) for the acquisition of thermotolerance in flowering plants^15–17^. In tea, HSPs, transcription factors (TFs and HSFs), calcium-binding proteins, genes involved in jasmonic and ascorbic acid pathways have been associated with heat stress regulation^18–21^. Recently, multiple studies have reported the key role of HSPs in sensing and signaling heat stress^22^. The use of HSP-specific inhibitor assay such as geldanamycin (GDA) has been reported with the activation of HSR in various plant species^23,24^. Moreover, rapid and simple leaf disc mediated inhibitor assay to study biotic/abiotic stress tolerance has been successfully used in multiple studies in plant system^25–27^. Being a polygenic trait, heat stress tolerance is controlled by various molecular components critical at different developmental stages/or plant tissues exhibiting spatio-temporal regulation of thermotolerance^28^. In addition, the genomic complexities in plants may provide an innate ability to sustain in harsh environmental conditions by modulating transcriptional programming to regulate a “complex biological network”^29^. Considering the limited information on tea plant response to a higher temperature, recently published draft tea genome is expected to facilitate better identification and characterization of heat stress-associated pathways in tea^30–32^.

Therefore, the current study aims to unravel the molecular programming regulating HSR by correlating the morphological, physiological and RNA-Seq expression analysis in tolerant and sensitive tea cultivars. The phenotypic screening based on thermotolerance response in 20 tea cultivars affirmed the contrasting response to heat stress. The study identified the key role of chaperons (HSP) and HSFs in inducing thermotolerance by regulating various thermo-responsive genes. Moreover, their correlation obtained with multiple metabolic pathways including tea quality, water regulation and starch metabolism in the predicted interactome transcriptional network indicates their direct/indirect role in the mitigation of heat stress. Furthermore, structure-based molecular docking of identified key HSP (*Cs*HSP90) followed by leaf disc inhibitor assay using GDA further supported their key role in inducing thermotolerance in tea. The current study, extrapolates a deeper understanding of chaperons mediated regulation of HSR in tea.

## Results

### Phenotypic response of tea cultivars under heat stress

**Fig. 1:**
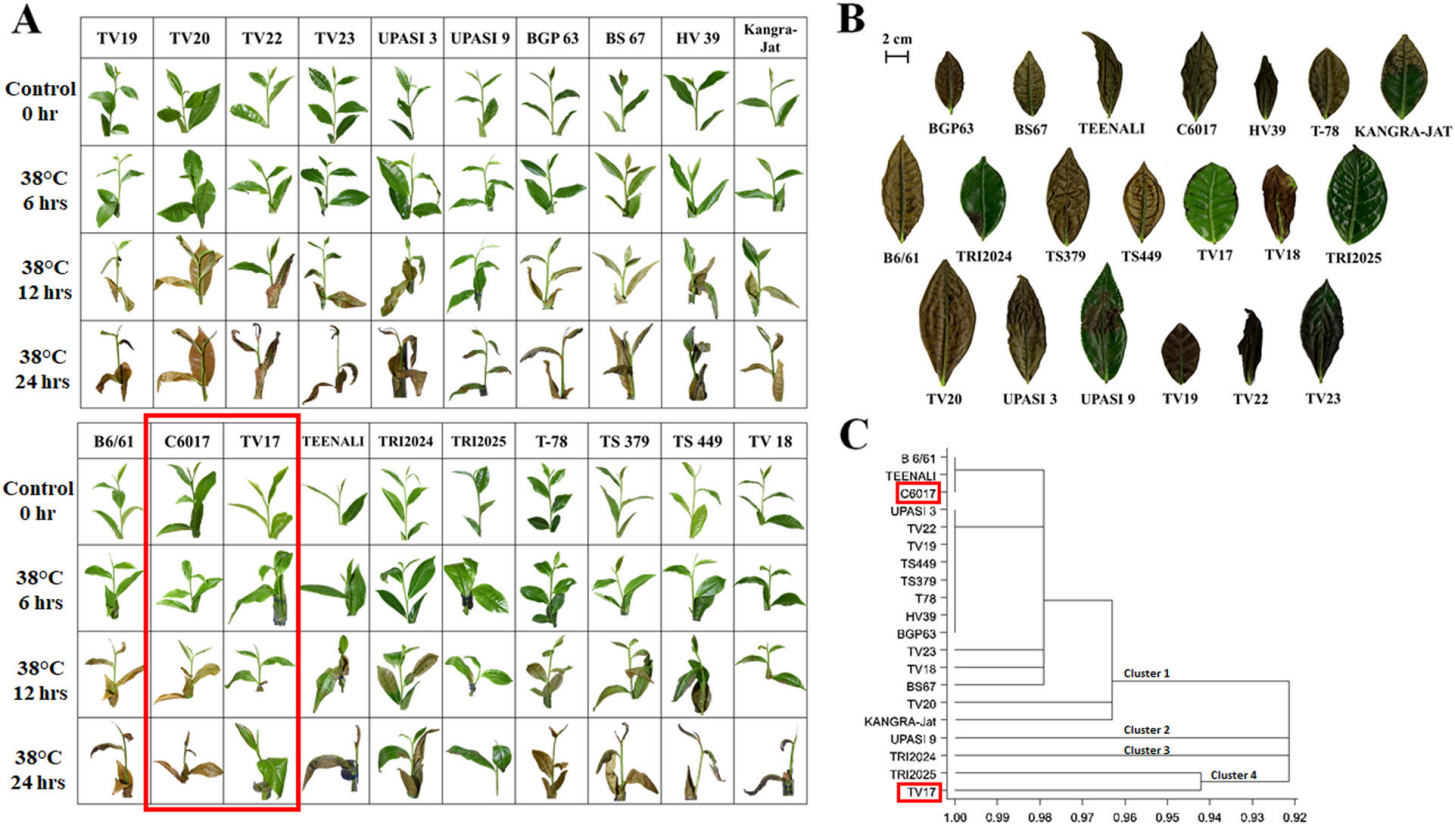
Heat stress response in tea. Heat tolerance evaluation of 20 cultivars based on scorching effects in plant (**A**) twigs and (**B**) third mature leaves; **C** Dendrogram based on Euclidean distance of qualitative score (scorching effect) clustering 20 cultivars in 4 clusters representing sensitive cultivars (cluster 1), moderately tolerant (cluster 2 and 3), and highly tolerant cultivars (cluster 4)

Twenty popular tea cultivars were subjected to heat stress for 0 h, 6 h, 12 h, and 24 h and scored for scorching effects using a five-point scale. Significant noticeable response to heat stress was recorded after 12 h of heat treatment. The tolerance and sensitivity of the cultivars were determined based on the level of leaf scorching, wherein, a heat-tolerant cultivar exhibiting ≤10% scorching of tissue with prolific flushing and no dormant shoots (score: 1); while heat-sensitive cultivar showing ≥76% scorching with severe leaf defoliation and completely dormant shoots (score 5). Cultivar TV17 recorded with less than 10% scorching effect (score: 1), while TRI2025, TRI2024, and UPASI-9 exhibited moderate tolerance with 11–25% scorching effect (score: 2). Moreover, the majority of the cultivars exhibited 26–50% scorching with dormant shoots, wilted and falling leaves (score: 3) to severe leaf defoliation in C6017 (score 5). However, by the end of the heat treatment, severe defoliation with multiple numbers of dead tissues was recorded in the majority of the cultivars (Fig. 1A, B). Subsequently, cluster analysis based on euclidean distance of qualitative score grouped 20 cultivars into four main clusters distinguishing, sensitive cultivars (cluster 1), moderately tolerant (cluster 2 and 3), and highly tolerant cultivars (cluster 4) (Fig. 1C). Based on this, cultivar TV17 was categorized as the most heat tolerant (HT), while, C6017 was the most heat-sensitive (HS) cultivar used for downstream elucidation of physiological and molecular insights of heat stress response in tea.

### Leaf stress injury

#### Heat stress negatively affects the leaf water content, oxidation potential, chlorophyll and increases membrane damage

**Fig. 2:**
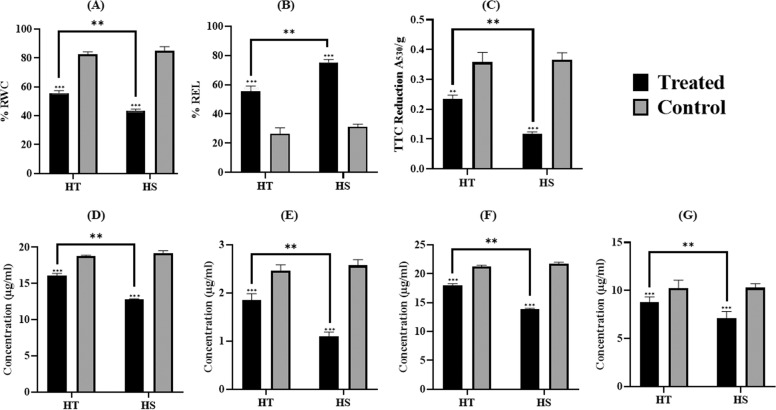
Physiological response under heat stress in tea. **A** Relative water content (RWC); **B** Relative electrolyte leakage (REL); **C** Cellular oxidizing ability using TTC reduction ability in leaves; **D** Estimation of chlorophyll a; **E** chlorophyll b; **F** total chlorophyll; **G** carotenoids in leaves. The data represents mean ± Standard deviation for replicated samples and the level of significance is represented by symbols (“***”, “**”, “*”) <=> *p*-values (0.001, 0.01, 0.05). The “HT” in the graph represents heat-tolerant cultivar and “HS” represents heat-sensitive cultivars

The water retention ability (relative water content: RWC) was observed to be negatively correlated with exposure to heat stress (Fig. 2A). However, significantly, higher RWC was recorded in the tolerant cultivar (55.7%) as compared to sensitive cultivar (47%), wherein, percentage of water loss relative to control was 41.8% and 27% in heat sensitive (HS) and heat tolerant (HT) cultivars, respectively. The electrolyte leakage determining the membrane damage in the leaf was remarkably increased on administrating heat stress (Fig. 2B). The sensitive cultivar recorded severe leaf injury due to higher electrolyte leakage (76.4%) than the tolerant cultivar (55.34%). Moreover, the cellular respiration (cellular oxidizing potential) estimated using TTC reduction revealed a significant decrease in the oxidizing potential during heat stress, which was relatively on a higher side in tolerant (65.5%) than sensitive cultivar (32.48%) (Fig. 2C). The Chlorophyll content seems to be negatively affected by heat stress, wherein, sensitive cultivars recorded higher chlorosis with a decrease in total chlorophyll content (33.7%) as compared to tolerant cultivar (10.99%) (Fig. 2D–F). Similarly, total carotenoid content was also reduced to a higher extent in sensitive than tolerant tea cultivar during the heat stress (Fig. 2G).

### Comparative transcriptional response to heat stress

#### Quality filtering, assembly, and functional annotation

The comparative transcriptome sequencing of control and heat-treated samples of sensitive and tolerant tea cultivars resulted in 42 million raw reads. Quality filtering and CSS genome-guided de novo assembly^31^ of 38 million filtered reads lead to 52,588 contigs with minimum sequence length and N_50_ of 300 bp and 1408 bp, respectively. Further, clustering resulted in 50,441 unigenes encapsulating 33,898 genes and 16,543 isoforms. Functional annotation with various publicly available databases annotated 37,509 (nr), 31353 (pfam), 29,181 (InterProScan), 28,607 (swissprot), 35,178 (TAIR10), 20,115 (TF) with 34,894 gene ontologies (GO) and 8954 KEGG pathway databases (Supplementary Table S1). The raw reads obtained were submitted to the NCBI SRA database under the bioproject PRJNA520786 with SRA accession number SRR12089312, SRR12089313, SRR12089314, and SRR12089315.

#### Differential gene and GO enrichment analysis

**Fig. 3:**
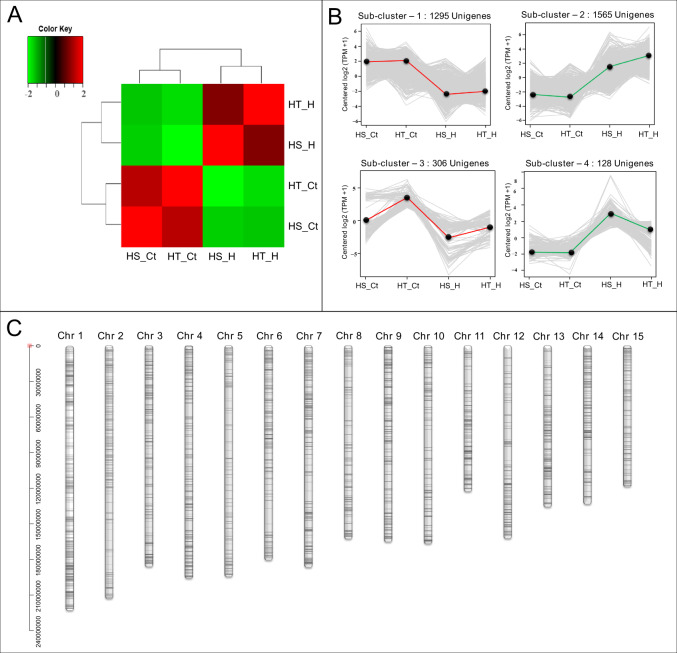
Clustering and chromosomal distribution of significant differentially expressed unigenes. **A** Correlation plot of significantly differentially expressed unigenes based on their median TPM values (FC ≥ 2, FDR < 1e−4); **B** Clustering of significantly differentially expressed unigenes based on median TPM values considering FC ≥ 2, FDR < 1e−4; **C** Distribution of significant differentially expressed 2831 unigenes on 15 tea chromosomes of tea

Overall, 3294 unigenes exhibiting significant differential expression (fold change, FC: ≥ 2; false discovery rate, FDR :< 1e−4) in both de novo and reference-based differential expression analysis were identified and grouped into 4 major clusters representing upregulated (Cluster 2 and 4) and downregulated (Cluster 1 and 3) unigenes (Fig. 3A, B; Supplementary Table S2). Subsequently, 2831 heat stress-associated unigenes exhibiting wider distribution across all the 15 chromosomes of CSS tea genome^32^ were considered for downstream gene/pathways enrichment analysis (Fig. 3C; Supplementary Table S2).

**Fig. 4:**
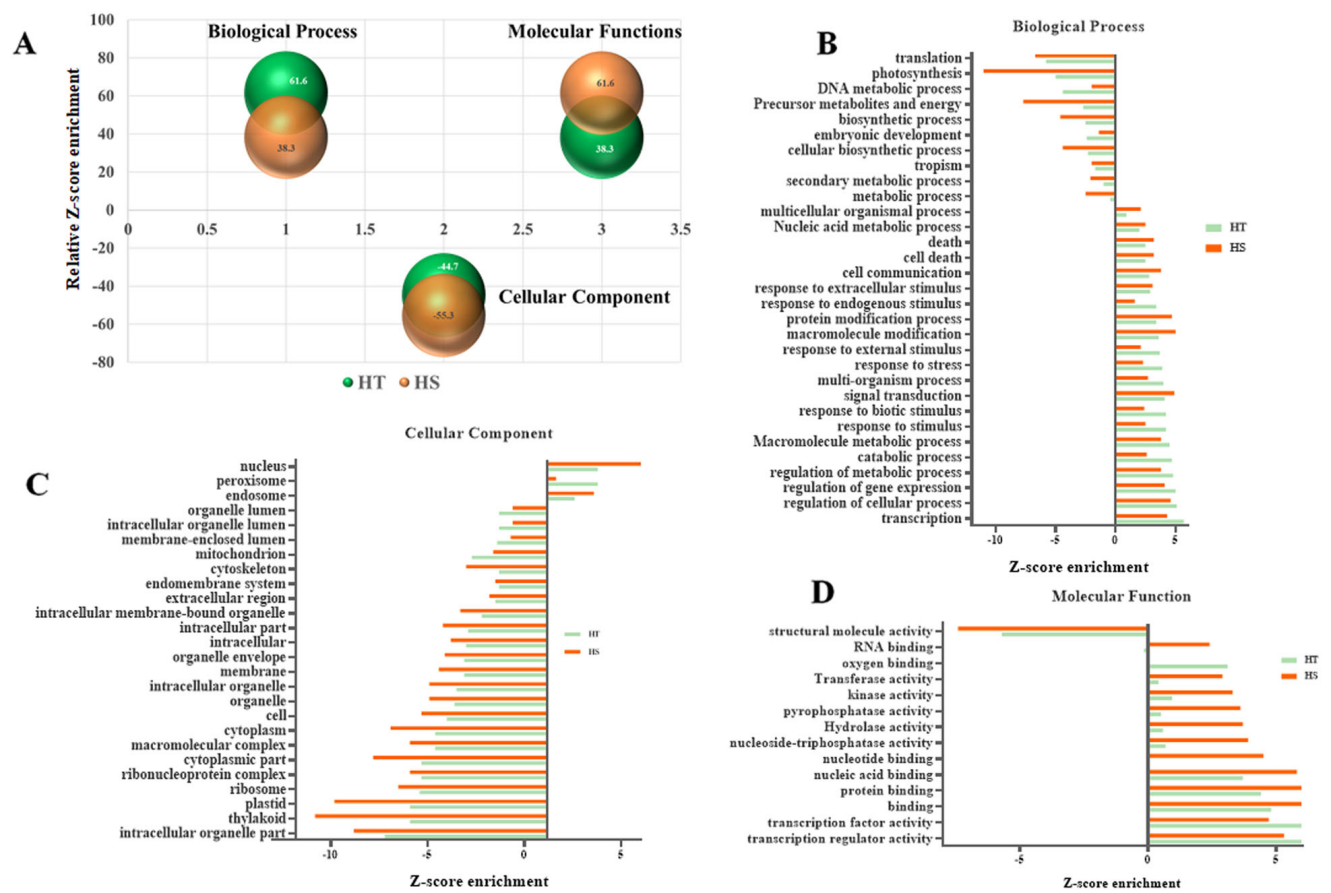
Differential enrichment of Gene ontology (GO) during heat stress. **A** Relative Z-score enrichment of GO terms corresponding to biological process, cellular component, and molecular functions represented by bubble plot in HT and HS cultivars; **B** Bar chart representing significant differential enrichment of Biological processes; **C** Cellular components and **D** Molecular functions in HT and HS cultivar

The comparative GO enrichment analysis recorded significantly higher enrichment of biological processes in the tolerant cultivar, while the molecular functions in the sensitive cultivar. However, the cellular components were negatively enriched in both the cultivars under heat stress with more significant in sensitive cultivar (Fig. 4A–D).

Under the category of biological processes significantly negative enrichment of photosynthesis (GO:0015979), translation (GO:0006412), cell cycle (GO:0007049), generation of precursor metabolite and energy (GO:0006091), and cellular biosynthetic process (GO:0044249) was recorded during the administration of heat stress in both tolerant and sensitive cultivar, nevertheless, more downregulated in the sensitive cultivar (Supplementary Fig. S1 and Supplementary Table S3A). While death (GO:0016265) and cell death (GO:0008219) were enriched significantly higher in sensitive cultivar [Z-score (HS: 3.2; *p*-value ≤ 0.01)], hence may be corroborated with intensive scorching effect in leaf. The enrichment of transcription (GO:0006350), regulation of cellular processes (GO:0050794), regulation of gene expression (GO:0010468), response to endogenous stimulus (GO:0009719), and response to external stimuli (GO:0009605), in the tolerant cultivar, indicate the lower effect of heat stress with efficient metabolic activity. These include key transcription factors *viz* HSFs; mitogen-activated protein kinases: MAPKs, WRKY domain, basic helix loop helix: bHLH, ethylene response factors: ERF, and basic leucine zipper: bzip having an indispensable role in regulating gene expression under heat stress.

During this study, unigenes corresponding to various cellular components exhibited significantly negative enrichment during heat stress (Supplementary Fig. S2 and Supplementary Table S3B). The GO terms specifically involved in membrane (GO:0016020), plastid (GO:0009536), thylakoid (GO:0009579), cytoplasm (GO:0005737), and ribosomes (GO:0005840) exhibited higher negative enrichment (*p-*value ≤ 0.001) under heat stress in sensitive tea cultivar. However, the peroxisomes (GO:0005777) and microbody (GO:0042579) reportedly involved in detoxification of ROS system under heat stress exhibited significantly higher positive enrichment in tolerant cultivar [Z-score (HT: 2.6; *p-*value ≤ 0.05)]. These include unigenes with jasmonate domain protein (JAZ), acyl activating enzyme, 3-ketoacyl-CoA thiolase (*KAT2*), and acyl CoA oxidase (*ACX*) involved in jasmonic acid synthesis during heat stress.

In the molecular function, transcription factor (GO:0003700), transcription regulator activity (GO:0030528), and oxygen binding (GO:0019825) were significantly enriched in tolerant cultivar (*p-*value ≤ 0.001), while, hydrolase (GO:0016817), transferase (GO:0016772) and kinase activity (GO:0016301) recorded with higher enrichment in sensitive cultivar (*p-*value ≤ 0.01). However, structural molecular activity (GO:0005198) was reduced significantly [Z-score (HT: −5.4; HS: −7.4); *p*-value ≤ 0.001] in both the cultivars during heat stress (Supplementary Fig. S3 and Supplementary Table S3C).

#### Differential pathways enrichment analysis

The pathway analysis exhibited crucial implications for the elucidation of complex physiological processes. Significantly negative enrichment of KEGG pathways (FDR ≤ 0.001), such as photosynthesis, antenna proteins involved in a light reaction, chlorophyll biosynthesis, and secondary metabolic pathways (flavonoid and phenylpropanoid biosynthesis) irrespective to sensitive and tolerant cultivars have well corresponded with GO enrichment inferences during heat stress (Table 1). The primary metabolic pathways including carbon metabolism, pyruvate metabolism, glycolysis, and carbon fixation in photosynthetic organisms exhibited higher enrichment in the tolerant cultivar. These include plant haem oxygenase family protein (*HMOX1*), Cyt c oxidase (*Cyt. C*), NADH dehydrogenase, and oxidoreductase involved in plasma membrane and mitochondrial respiratory electron chain complex. The genes corresponding to flavonoid pathways such as chalcone synthase (CHS), chalcone isomerase (CHI), leucoanthocyanidin reductase (LAR), anthocyanidin reductase (AR), and flavonoid hydroxylase (FH) were recorded with significant downregulation in sensitive cultivar during heat stress. In addition, the plant hormone signal transduction pathways [ath04075; Z-score (HT: 2.5; HS: 0.9); FDR ≤ 0.01], MAPK signaling [ath04016; Z-score (HT: 2.4; HS: 1.3); FDR ≤ 0.01] and secondary metabolite biosynthesis pathways [ath01110; Z-score (HT: 1.9; HS: 0.9); FDR ≤ 0.05] were found with significantly higher positive enrichment during heat stress in tolerant cultivar. On the contrary, glutathione metabolism, autophagy, and alpha-linolenic acid metabolism exhibited significantly higher enrichment in the sensitive cultivar.

**Table 1:** Significantly differentially enriched KEGG pathways in tolerant and sensitive cultivars during heat stress in tea

S. No	Pathways ID	Enriched Pathways	HT		HS		FDR
			Unigenes mapped	Z-score	Unigenes mapped	Z-score	
1	ath01200	Carbon metabolism	92	1.1	65	0.9	0.0156
2	ath00941	Flavonoid biosynthesis	10	−3.7	10	−4.5	0.00017
3	ath00940	Phenylpropanoid biosynthesis	26	−1.7	25	−2.5	0.0378
4	ath00195	Photosynthesis	18	−3	6	−5.2	4.33E−05
5	ath00196	Photosynthesis—antenna proteins	12	−2.8	6	−4.4	0.00046
6	ath00860	Porphyrin and chlorophyll metabolism	11	−0.4	11	−2.4	0.0062
7	ath04141	Protein processing in the endoplasmic reticulum	39	1.5	39	0.5	0.0381
8	ath04120	Ubiquitin mediated proteolysis	58	4.1	58	2.2	0.0405
9	ath03050	Proteasome	25	2.5	11	1.2	0.0154
10	ath00592	alpha-Linolenic acid metabolism	12	2.9	11	3.1	0.000046
11	ath04136	Autophagy—other	18	1.4	18	2.6	0.0035
12	ath01110	Biosynthesis of secondary metabolites	196	1.1	196	0.9	0.0129
13	ath00710	Carbon fixation in photosynthetic organisms	11	3.7	20	1.9	0.0035
14	ath00480	Glutathione metabolism	20	1.4	20	2.2	0.0378
15	ath00010	Glycolysis/gluconeogenesis	27	1.5	27	1	0.01041
16	ath04016	MAPK signaling pathway—plant	41	2.4	41	1.3	0.0035
17	ath00360	Phenylalanine metabolism	10	1.8	10	1	0.0105
18	ath04075	Plant hormone signal transduction	68	2.5	68	0.9	0.0083
19	ath00620	Pyruvate metabolism	34	2.1	34	1.2	0.0289

#### Prediction of heat stress associated key pathways in tea

**Fig. 5:**
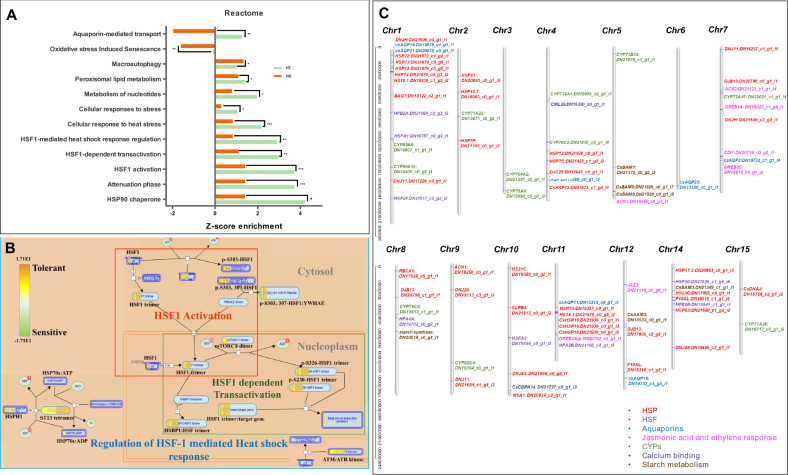
Heat stress associated pathways enrichment. **A** Bar chart representing significant differential enrichment of heat stress associated pathways in HT and HS cultivar using reactome database. **B** HSF pathway representing significant upregulation of HSF1 activation, HSF1-dependant transactivation, and HSF1 mediated heat shock response regulation pathways. The scale represents enrichment score wherein positive value denotes upregulation in tolerant, while negative denoted upregulation in sensitive. **C** Distribution of chaperons (Heat shock proteins), heat shock transcription factors (HSFs), aquaporins, Jasmonic acid, and ethylene response, CYPs calcium-binding, and starch metabolism-related unigenes to 15 chromosomes in tea

The specific heat stress-associated pathways were curated using high-performance reactome pathway enrichment analysis. The study revealed significantly higher enrichment (FDR ≤ 0.05) of “cellular response to heat stress” including “HSP90 chaperon”, “attenuation phase”, “HSF1 activation”, “HSF1 dependant transactivation”, “HSF1 mediated heat shock regulation” and “peroxisomal lipid metabolism” under heat stress (Fig. 5A, B). These majorly include HSPs, peroxidases, catalases, dehydrin, coronatine insensitive (COI1), metalloprotease-like functions (ATP dependent zinc metalloprotease and DNA dependent metalloprotease), RBL14 (RHOMBOID like protein 14), and LEA (late embryogenesis abundant proteins) exhibiting significant upregulation in tolerant cultivar. The HSPs are known to play a key role in providing thermal stress (TS) tolerance in plant species, which was also evident with the data obtained in our study. These include 11 unigenes with DNAJ domain, (8) HSP70 protein family, (4) unigenes with HSP90 domain, (3) HSP70-interacting protein, (18) low molecular weight HSPs and (7) HSFs with higher upregulation in the tolerant cultivar. In addition, chromosomal mapping identified the genome-wide distribution of HSPs and HSFs (except *Chr 3, Chr 5*, and *Chr 6*) with the highest distribution in *Chr 1* (Fig. 5C).

#### Aquaporin mediated heat stress tolerance

“Aquaporins”, the membrane channel proteins are known to play a key role in abiotic stress tolerance in plants^33^. The unigenes with aquaporins domains of plasma membrane intrinsic protein (PIP) family (*Cs*AQU11, *Cs*AQU15, *Cs*AQU17, *Cs*AQU18, and *Cs*AQU19) involved in “aquaporin-mediated transport” were upregulated in the tolerant cultivar. Nevertheless, the tonoplast intrinsic protein family (*Cs*AQU21) was significantly downregulated during heat stress irrespective of tolerant and sensitive cultivars. Furthermore, genome-wide assignment of aquaporins involved in thermotolerance was majorly distributed in *Chr* 1, 4, 6, 7, 11, and 12 of CSS tea genome (Fig. 5C).

#### Starch metabolism and calcium signaling

The unigenes having strong homology with genes involved in starch metabolism including beta-amylases (*Cs*BAM1, *Cs*BAM3, and *Cs*BAM9) and alpha-amylases (*Cs*AAM1 and *Cs*AAM3) assigned to *Chr 5, 12*, and *14*, recorded significantly higher upregulation in the tolerant cultivar (Fig. 5C). Similarly, calcium dependant protein kinases (*Cs*CDPK14) and calcineurin-like domains reportedly involved in heat stress tolerance, also exhibited significantly higher upregulation tolerant cultivar. Nevertheless, hypothetical unigenes having a calmodulin-like domain exhibited significant downregulation during heat stress irrespective of tolerant and sensitive cultivars.

#### CYPs and ethylene signaling

Interestingly, 23 unigenes encoding CYPs, with wider distribution across the tea genome (*Chr 1, 2, 3, 4, 5, 7, 8, 9, 15)* were significantly upregulated during heat stress and exhibits higher enrichment of 17 unigenes (greater than twofold) in tolerant cultivar (Fig. 5C). Similarly, unigenes corresponding to the ethylene signaling pathway in our study seems to be upregulated during the administration of heat stress. The ethylene response factors (ERFs transcription factor) and ethylene insensitive-like (EIN) were significantly upregulated in the tolerant cultivar, while, ethylene receptor proteins involved in ethylene signal transduction were upregulated in the sensitive cultivar.

#### Heat stress associated interactome network analysis

**Fig. 6:**
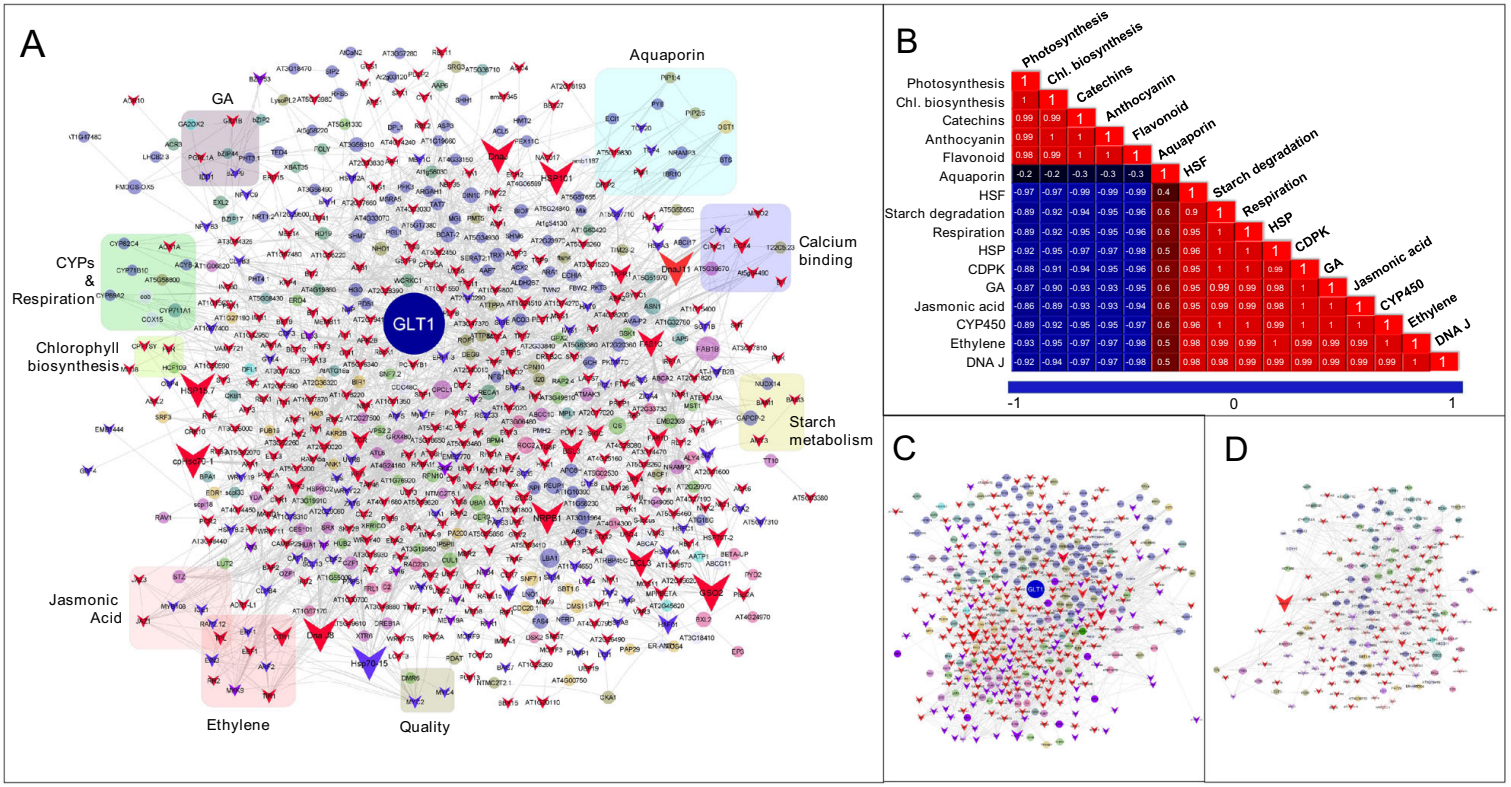
Heat stress associated transcriptional interactome network prediction in tea. **A** Predicted interactome network showing direct/indirect interactions of Heat shock proteins (HSPs) and Heat shock transcription factors (HSFs) (represented with inverted arrows) with various physiological pathways (**B**) Pearson’s correlation of enriched pathways in the predicted network. Enriched HSPs and HSFs in (**C**) tolerant and (**D**) sensitive cultivars

To comprehensively infer the heat stress associated pathways, the transcriptional interactome network was predicted using significantly differentially expressed (DE) unigenes in HS & HT. A network comprising of 639 genes (nodes) with 2613 edges was built with an average number of neighbors 7.984 and a clustering coefficient of 0.128 (Fig. 6A; Supplementary Table S4). The interactions of HSPs and HSFs with unigenes actively involved in respiration, chlorophyll biosynthesis, gibberellic acid, jasmonic acid, and ethylene biosynthesis, aquaporins, calcium-binding, and starch metabolism suggests their involvement in regulating a wide range of physiological pathways during heat stress in tea. In addition, Pearson’s correlation network built using gene expression pattern recorded a positive correlation of heat stress associated genes (HSP, HSFs, and DNAJ) with aquaporins, CDPKs, genes associated with starch degradation, gibberellic acid synthesis, jasmonic acid, cellular respiration, and CYPs (Fig. 6B). However, unigenes involved in photosynthesis, chlorophyll biosynthesis, and attributing tea quality (anthocyanins, flavonoids, and catechins) were negatively correlated with heat stress-associated unigenes in the predicted network.

Significantly higher enrichment of heat stress-associated unigenes was recorded in the major hub of tolerant cultivars (458) in contrast to sensitive cultivar (199) (Fig. 6C, D; Supplementary Table S5). These include TFs (*Cs*HSFA2, HSFB2A, HSFC1, HSPRO2, WRKY, MPK3, and MBF1C), HSP chaperons (*Cs*HSP90, *Cs*HSP70, HSP101, and *Cs*HSP17.6) and co-chaperons (ROF1, ROF2, CLPB3, BAG7) possibly associated with thermotolerance by inducing HSR in tolerant cultivar. The direct interactions of *Cs*HSP90 with *Cs*HSFA2, *Cs*HSP70, and *Cs*HSP17.6 indicate its key role in inducing HSR in tea. Moreover, the indirect interaction of *Cs*HSP70 with aquaporins (*Cs*AQU15 and *Cs*AQU19) and trehalose phosphate synthase with a strong positive correlation in their expression pattern suggests their role in water channel regulation during heat stress. In addition, direct interactions of HSPs and co-chaperons (DNAJ) with CYP450 and some essential unigenes involved in the cellular respiration [glyceraldehyde 3 phosphate dehydrogenase, (GAPCP-2), cyt c oxidase (COX), cyt b reductase (cob), and NADPH dehydrogenase quinone reductase like protein], indicates shifting of other metabolic activities to essential primary metabolic activity for enduring the heat stress. Furthermore, indirect interactions obtain between heat stress-associated genes with ethylene signaling pathways (ERFs, EIN, and Auxin Response factors, ARFs) and starch metabolism (*Cs*BAM1 and *Cs*BAM3) indicate their association in thermotolerance by regulating starch degradation during heat stress.

#### RNA-seq data validation by qRT-PCR

**Fig. 7:**
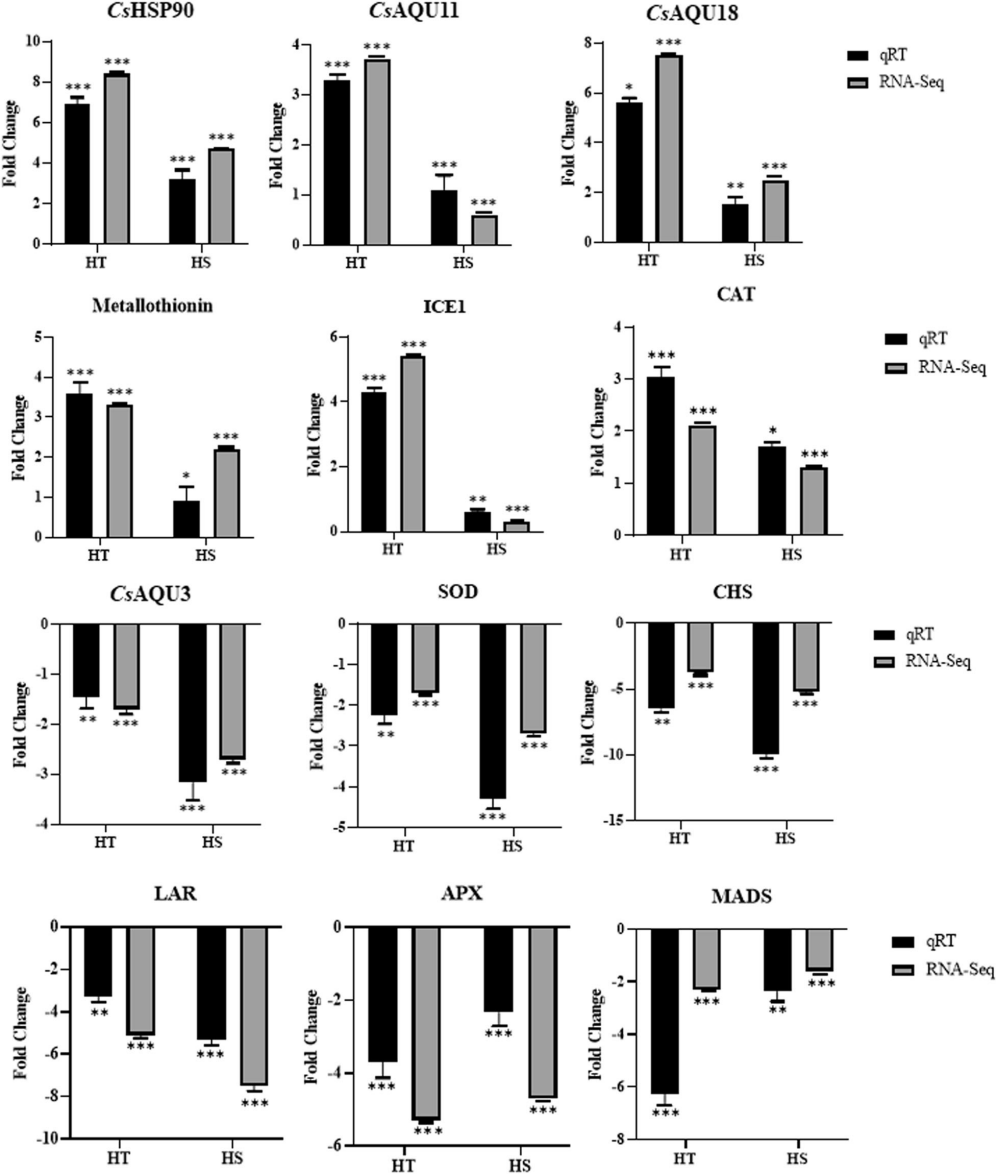
qRT-PCR based RNA-Seq DEGs validation of 12 selected genes having a significant role in heat stress. A strong positive Pearson’s correlation (*R*^2^ = 0.79) obtained between significant DEGs and RNA-Seq data which indicates higher reliability of RNA-Seq data. The bar represents SD of fold change for three replicated experiments and symbols (“***”, “**”, “*”) represent significance corresponding to *p*-values (0.001, 0.01, 0.05)

The differential expression analysis from RNA-Seq data was well corroborated with quantitative real-time PCR analysis of 12 key genes involved in heat stress (Fig. 7). The heat stress-associated genes including HSPs (*Cs*HSP90), aquaporin (*Cs*AQU11, *Cs*AQU18), catalase (CAT), ascorbate peroxidase (APX), metallothionin1, and ICE1 were recorded higher expression with significant upregulation in the tolerant cultivar during heat stress. However, unigenes attributing to tea quality (CHS; LAR), along with transcription factor (MADS), and *Cs*AQU3 were downregulated during heat stress.

#### Structural modeling and docking of *Cs*HSP90

**Fig. 8:**
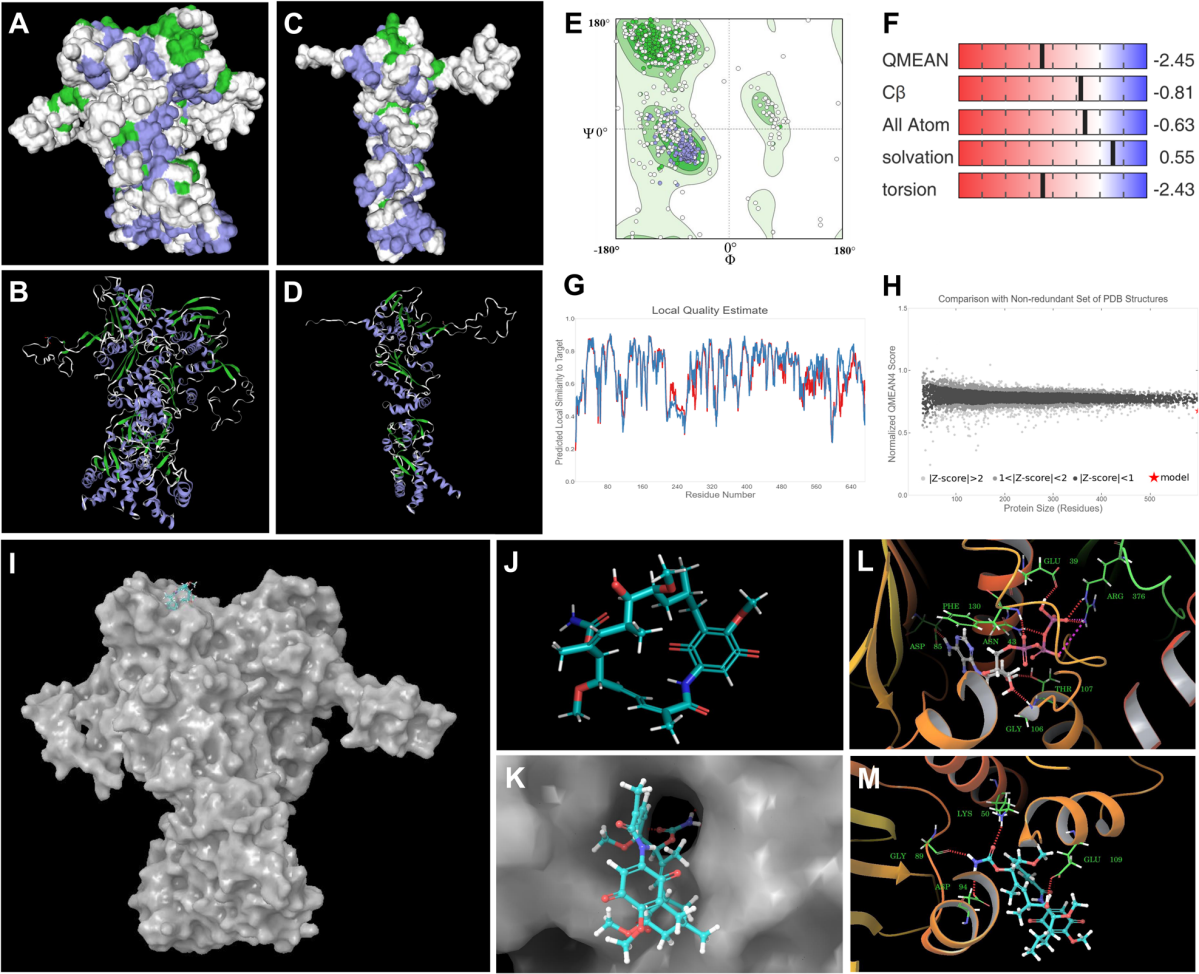
3D structural modeling and docking of DN21680_c1_g2_i2 with GDA. **A** Predicted 3D model of DN21680_c1_g2_i2 homodimer using Endoplasmin (GRP94) template represented in surface; and (**B**) helix loop helix format. **C**, **D** Monomer (Chain B) of the homodimer. **E** Ramachandran plot of predicted structure 92.41% amino acid sequences in the allowed region, (**F**–**H**) QMEAN score and local quality estimate of the predicted model. **I** Surface model of predicted protein with docked GDA, **J** molecular structure of geldanamycin (MF: C29H40N2O9; MW: 560.6 g/mol). **K** Blocking of ATP binding pocket of the predicted model by GDA. **L** Bound ATP with protein residues. **M** Binding of GDA molecule with the amino acid residues Lys50, Gly89, Asp94, and Glu109 of predicted *Cs*HSP90 protein structure

Considering the key regulatory role of *Cs*HSP90 during HSR as evident with significant enrichment of “HSP90 chaperon” in reactome pathway and predicted interactome network, *Cs*HSP90 unigene (DN21680_c1_g2_i2) was selected for downstream structure prediction and docking with GDA in tea. As the inhibitory role of GDA to *Cs*HSP90 protein has not been studied in tea, hence, in silico structure prediction and molecular docking were performed to demonstrate GDA mediated inhibition of *Cs*HSP90. The sequence-based phylogenetic analysis recorded its closest homology with cytosolic HSP90 in *Arabidopsis thaliana* and *Hordeum vulgare* (Supplementary Fig. S4). Moreover, the predicted 3D structure of *Cs*HSP90 comprises of homo-dimer belonging to family HSP90 of histidine kinase-like ATPase superfamily, wherein, each homodimer possesses three structural domains as N-terminal domain (NTD) (with ATP-binding site), middle domain (Client binding), and C-terminal domain involved in dimerization (Fig. 8A–H; Supplementary Table S6).

Subsequently, molecular docking interaction between the predicted 3D model of *Cs*HSP90 with GDA indicates its blocking property at ATP binding pocket near NTD of both chains A and B (Fig. 8I–M). This property was characterized by the binding of ATP molecule with the residues Glu39, Phe3, Asp85, Asn43, Thr107, and Gly106 of the predicted protein. The lower binding energy (−30.502 Kcal/mol) of GDA with the residues Lys50, Gly89, Asp94, and Glu109 indicates its stronger binding affinity with the predicted protein model (*Cs*HSP90). Hence, the docking results with GDA indicate its blocking effect in ATP binding sites of predicted *Cs*HSP90 structure, probably affecting its ATP dephosphorylation efficiency.

#### Leaf disc inhibitor assay to assess *Cs*HSP90 chaperon activity

**Fig. 9:**
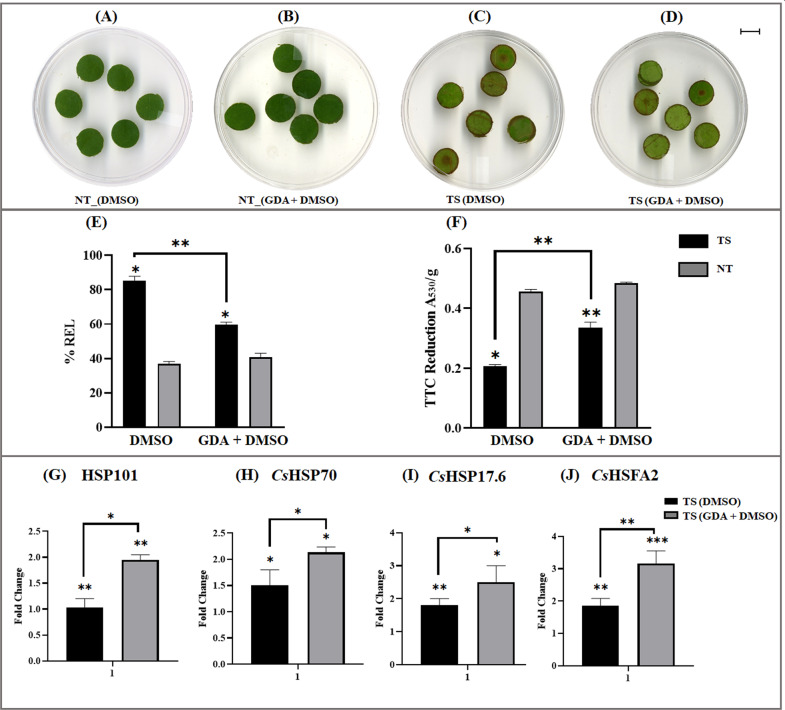
Leaf disc assay with HSP90 inhibitor in tea. **A**, **B** Leaf disc under normal temperature (NT) in (**A**) DMSO solution without GDA and (**B**) DMSO + GDA solution. **C**, **D** Leaf disc after 24 h of high-temperature treatment (thermal stress: TS) in (**C**) DMSO solution (without GDA) and (**D**) DMSO + GDA solution. **E** Relative electrolyte leakage and (**F**) cellular oxidation ability using TTC reduction ability of leaf disc in DMSO and GDA + DMSO solution during NT and TS conditions. **G**–**J** Relative expression (fold change) of HSP family proteins on leaf samples with and without GDA treatment. The bar represents SD of fold change using three replicates while symbols (“***”, “**”, “*”) represent the level of significance at *p*-values (≤0.001, 0.01, 0.05)

Considering the inhibitory role of GDA as observed in structure-based molecular docking of *Cs*HSP90, GDA mediated HSP90 inhibitor assay was used to validate their functional role in HSR in tea^23^. Slight morphological changes with decreased scorching in the leaf disc of tea cultivar C6017 (HS) were observed in the presence of GDA (Fig. 9A–D). Interestingly, a significant reduction in relative electrolyte leakage (REL) (21.9%) along with an increase in cellular oxidation potential (23.6%) was recorded in GDA treated leaf disc (dimethyl sulfoxide (DMSO) + GDA) as compared to untreated leaf disc (only DMSO) under TS conditions (Fig. 9E, F). However, no significant differences in REL and cellular oxidizing potential were observed between the control samples (DMSO) under normal temperature and TS conditions. In addition, the quantitative expression analysis using qRT-PCR recorded a significant upregulation of small HSPs (*Cs*HSP17.6), high molecular weight *Cs*HSP70, HSP101, and HFSs (*Cs*HSFA2) in presence of GDA, probably may correspond to transcriptional activation of HSR response in tea (Fig. 9G–J). Nevertheless, no significant change in responses was recorded in cultivar TV17 (HT), which may be due to its inherent ability to withstand heat stress.

## Discussion

The gradually increasing temperature in most of the tea growing regions is associated with a subsequent decline in tea production (yield) and quality with increase in plant mortality^9,10^. Therefore, the development of improved (high yield and quality) climate-resilient tea cultivars remains a key breeding targets. To achieve this, a comprehensive understanding of the system biology of plant response to various abiotic stress is required. Under thermal stress (TS) conditions, a multitude of biological, physiological and molecular processes controlling plant growth are negatively affected^11^. In the current study, comparative high-throughput next-gen transcriptome sequencing was remarkably used to unravel key molecular insights controlling heat stress tolerance *vis-a-vis* correlating the differentially expressed key genes with morphological and physiological attributes in tea^1,4,5^. To get the global response to high-temperature stress, an evaluation of 20 tea cultivars revealed differential phenotypic responses to heat stress, possibly due to inherent genetic diversity^34,35^. Interestingly, cultivars TV17 (HT) and C6017 (HS) selected to explicate physiological and molecular insights controlling heat stress response exhibited a similar trend under drought stress in tea^5^.

**Fig. 10:**
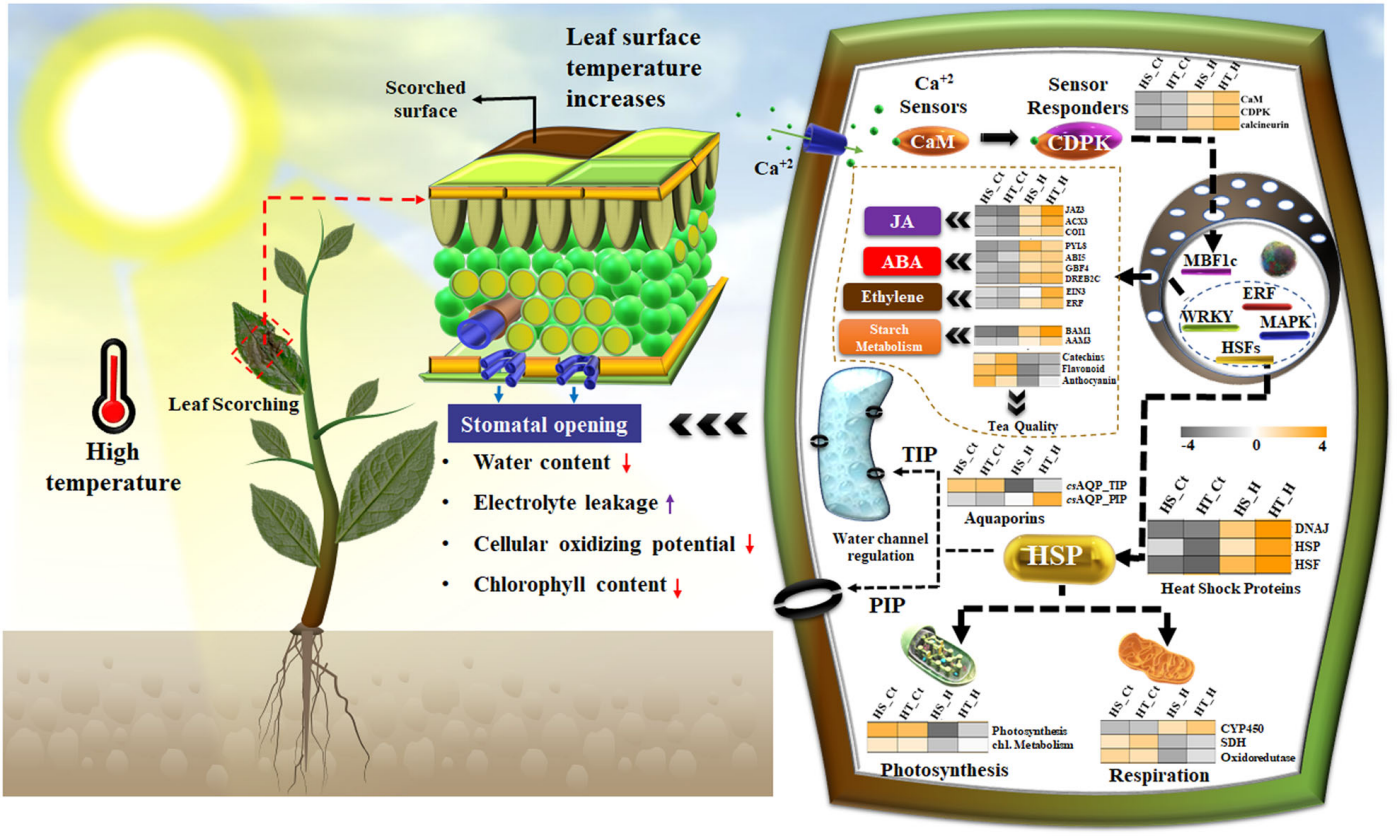
Summarized illustration of physiological and molecular insights underlying heat stress associated thermotolerance in tea. The higher temperature leads to an increase in leaf surface temperature with decreased water content, cellular oxidizing ability, and chlorophyll content, while an increase in the membrane damage due to high electrolyte leakage. The heatmap represents gene enrichment in heat sensitive (HS) and tolerant (HT) cultivars under control (HS_Ct; HT_Ct) and under heat treatment (HS_H; HT_H)

The tea plant with C3 photosynthetic mechanism is reported to be efficient at an optimum temperature of 25–30 °C^36,37^. However, the higher temperature (38 °C) used in this study seems to impair this mechanism in sensitive cultivar consecutively inhibiting cellular oxidizing potential (respiration rate) and stomatal conductance as reported in previous studies. Subsequently, this may enhance membrane damage resulting in a higher degree of scorching and chlorosis in tea. Moreover, the lower REL with higher leaf water content (RWC), chlorophyll content, and cellular oxidation potential in tolerant cultivar (Fig. 10), indicates its better ability to withstand TS complementing the previous report under water deficit condition in tea^6^.

Corresponding to derived morphological and physiological inferences, genome-wide transcriptional profiling further assisted us to decode the molecular programming distinguishing physiological response towards heat stress in tea. Integration of both references guided de novo assembly (33,898 genes and 16,543 isoforms; N50: 1408) and reference-based analysis with higher mapping rate (77.7%) to both CSS and CSA tea genome provided better resolution for identification of full-length key candidates underlying heat stress-associated metabolic pathways in this study^38–41^. Moreover, clustering of differentially expressed unigenes obtained from both de novo and reference-based differential expression analysis is advantageous to tackle both sample and technical biasness, thus improving the significance of our data as successfully demonstrated in the previous studies^1,5^. The differential GO, KEGG and plant reactome pathway enrichment analysis further supported our physiological inferences by exhibiting significant negative enrichment of photosynthesis (photosystem II) and quality-related secondary metabolic pathways. However, significantly higher enrichment of “HSP90 chaperon”, “HSF1 activation/transactivation”, “HSF1 mediated heat shock regulation” indicates an inverse relationship between heat stress mitigation and photosynthesis, wherein thylakoid (photochemical reactions) and stroma (carbon metabolism) seem to be an initial site of injury as reported in earlier studies in tea^42,43^. In addition, higher enrichment of “peroxisomes” and “microbody” along with “cellular response to heat stress” indicates enhancements of HSPs mediated ROS detoxification in tolerant cultivar, supporting previous studies in Arabidopsis, soybean, tobacco, and wheat species^44^. The aforementioned findings were also supported by the predicted interactome network, wherein identified key HSPs and HSFs exhibited direct/indirect interactions with important biological processes such as starch metabolism, phytohormones signaling, calcium signaling, aquaporin-mediated transport, ABA dependant/independent, and ethylene-response signal transduction. This possibly indicates well-co-ordinated HSPs and HSFs mediated regulation of biological processes, conferring thermotolerance in tea^18–21^. Moreover, genome-wide mapping of identified key candidates to different positions of CSS chromosomes, suggests their evolutionary history and role in influencing genetic variation of heat stress tolerance in tea^32^. The distribution of different gene families (aquaporins, starch metabolism, CYPs, calcium, and ethylene signaling) in chromosomes indicates their neighborhood connectivity, which may be involved in co-regulation as also recorded in our predicted interactome network^45^. In addition, the genome-wide localization of most of the HSPs towards the telomere region of chromosome (*Chr* 1, 8, 9, 10, and 14) suggests their potential role in improving thermotolerance in tea. Moreover, clustering of these HSPs to *Chr*1 and *Chr*11 suggests their evolutionary preferences^32^. Unlike previous studies, successful chromosomal mapping of key candidates will possibly accelerate the prediction of heat-stress-specific variants for expediting clonal selection in tea^32,40,46,47^.

In most plant species, the starch metabolism is considered a key determinant of plant-fitness during the abiotic stress, therefore upregulation of starch degrading enzymes (*Cs*BAM and *Cs*AAM) in the current study suggests their role in inducing sugar (maltose) accumulation for protection of ETC in photosynthesis^48^. Sugars released during starch degradation are known to act as both signaling molecules and osmoprotectants activating the ABA-dependent pathway to mitigate against negative effects of heat stress^49–51^. Direct/indirect interactions between identified HSPs /HSFs and key candidates associated with starch metabolism in the interactome network further affirmed their important role in regulating starch metabolism attributing thermotolerance in tea as reported in Arabidopsis^49^. In addition, the starch breakdown in guard cells is known to maintain the leaf surface temperature by regulating stomatal opening^48^. Therefore, strong positive enrichment of “aquaporin-mediated transport” in tolerant cultivar indicates their role in maintaining the plant water relationship during high temperature, which was substantially affected in sensitive cultivar leading to reduced water content and higher scorching effect^33^.

The enhanced phytohormone biosynthesis (beta-oxidation of jasmonic acid), WRKY, and MAPK signaling pathways exhibiting direct/indirect interactions with HSPs/HSFs in the current study suggest re-organization of protein/cellular structure for re-establishment of cellular redox balance and cellular homeostasis in tea^21,42,52^. Calcium ions, ubiquitous messengers in plant signaling were reported to an important role in reproduction and abiotic stress response in tea^1,53^. The significant upregulation of CDPKs and calcineurin-like domains in tolerant cultivar suggests an efficient role of calcium signaling pathways during heat stress in tea, wherein, CDPKs may act as “sensor responders” for decoding the calcium signal during abiotic stress response as reported in Arabidopsis^53^ (Fig. 10). The significant transcriptional interactions recorded between calcium signaling genes and HSFs (*Cs*HSFA1, *Cs*HSFA2, *Cs*HSFB2A) in current study, indicates CDPK mediated regulation of HSPs (*Cs*HSP90, *Cs*HSP70, *Cs*HSP17.6) to mitigate against heat stress in tea^19^.

Considering the highlighted role of HSPs as supported by significant enrichment and interaction with key biological processes involved in attenuating heat stress, *Cs*HSP90 unigene was successfully used for structure prediction and validation. The closest sequence homology with cytosolic HSP90 in *Arabidopsis thaliana* suggests its key role in inducing HSR by regulating HSFs in tea^24^. Moreover, structure-based molecular docking indicated GDA mediated inhibition of predicted cytosolic *Cs*HSP90 protein activity by blocking the ATP binding pocket at the NTD in tea. This may attribute to induced transcriptional activation of HSR cascade as recorded in HPS90 specific GDA mediated leaf-disc inhibition assay, exhibiting similar response reported in Arabidopsis and tomato^23,24,54^. Furthermore, significantly upregulated expression of *Cs*HSP17.6, HSP101, *Cs*HSP70, and *Cs*HSFA2 in GDA-treated leaf disks can be correlated with enhanced HSR^24^. Interestingly, significant interactions between *Cs*HSFA2 and HSPs including *Cs*HSP90, *Cs*HSP70, *Cs*HSP17.6, and HSP101 further support their potential role in inducing HSR, improving thermotolerance in tea. In addition, higher expression of HSF *Cs*HSFA2 may be attributed to transcriptional activation of heat-inducible genes enhancing HSR^24,54^. Furthermore, higher expression of *Cs*HSP17.6 (small molecular chaperon) probably regulating chloroplast membrane proteins dynamics *via* ROS scavenging in tea^18^. Moreover, upregulation of HSP101 might indicate thermo-memory under HSR as reported earlier in model plant^55^.

## Conclusion

The global warming has negatively affected the quality and yield potential of tea. Here combining morphological, physiological, and global transcriptional profiling will enrich heat stress associated functionally relevant genomic resources for genetic improvement of tea. Furthermore, key chaperons (HSPs and HSFs) identified here can be potential candidates to understand the mechanistic role of HSR and combining thermotolerance in high yielding quality tea cultivars. The coordinated transcriptional, reactome pathway enrichment, interactome network, 3D structural modeling, and docking along with inhibitor assay used successfully for functional characterization of key regulators of heat-shock response for the first time in this study, can be extrapolated for rapid dissection of desirable traits. The potential candidates identified in this study can be utilized for expediting the development of climate-resilient cultivars in tea.

## Materials and methods

### Plant materials

Twenty popular tea cultivars were screened for their phenotypic response to heat stress under controlled conditions (Supplementary Table S7). The 1-year-old vegetatively propagated tea plants were first acclimatized in a growth chamber with 16-h light (200 μmol m^−2^ s^−1^; 24 ± 1 °C), 8-h dark (19 ± 1 °C) photoperiod with a relative humidity of 70 ± 5% for 28 days. The plants were arranged in a randomly complete block design (RCBD) wherein three plants per cultivar replicated three times and subjected to 38 °C heat stress. The third mature leaf from the bud was used to determine the scorching effect at 0, 6, 12, and 24 h after treatment. A five-point scale was used for scoring the scorching effect to determine the tolerance/sensitivity level of each cultivar as previously described and data collected in three biological and technical replicates each (Supplementary Table S8)^56^. The mean score of each treatment per cultivar was used for the construction of neighbor-joining dendrogram based on euclidean distance using GenStat statistical tool *ver*. 15.1 (VSN International).

### Leaf stress injury

The stress injury in response to heat shock is evaluated in a third mature leaf of the most tolerant and sensitive cultivar after 24 h of heat treatment using RWC, electrolyte leakage, chlorophyll content, and triphenyl tetrazolium chloride (TTC) reduction^57^.

### RWC

The RWC was evaluated in heat tolerant (HT) and sensitive cultivars (HS) according to Barrs and Weatherley (1962) method^58^. Leaf tissue of 100 mg was collected and immersed in the distilled water for about 6 h and weighed (Turgid weight, TW) after surface drying. The dry weight (DW) was estimated after drying the leaves at 84 °C for 24 h in oven^6^. The RWC calculated using the following formula:

$${\mathrm{RWC}} = {\frac{{\mathrm{Fresh}\,\mathrm{Weight}\,\left( \mathrm{FW} \right) - \mathrm{Dry}\,\mathrm{Weight}\,\left( \mathrm{DW} \right)}}{{\mathrm{Turgid}\,\mathrm{Weight}\,\left( \mathrm{TW} \right) - \mathrm{Dry}\,\mathrm{Weight}\,\left( \mathrm{DW} \right)}}\times 100}$$

### REL for membrane damage

The permeability of the plasma membrane estimated by calculating REL. The leaf segments of one gram incubated in deionized water (10 mL) at room temperature overnight. The electrical conductivity was estimated after 24 h of incubation (before autoclaving: BA) and then autoclaved at 120 °C for about 20 min to measure final conductivity (after autoclaving: AA)^59^.

$${\mathrm{REL}}\,(\%) = {\frac{\mathrm{BA}}{\mathrm{AA}} \times \,100}$$

### Chlorophyll and carotenoid content

The chlorophyll content of tolerant and sensitive cultivar along with their respective controls was estimated as previously suggested^60^. One hundred milligrams leaf disc grounded into 4 mL of 80% acetone. The final volume was adjusted to 10 mL of acetone and the absorbance was measured at 665 nm, 645 nm, and 470 nm using spectrophotometer^61^. The concentration of chlorophyll and carotenoid estimated in µg/mL of acetone according to the following formula

$$\begin{array}{l}Chl\,a \, \left( {\mathrm{\mu g/mL}} \right) = {\it{12}}{\it{.25}}\,X\,A_{{\it{665}}}-{\it{2}}{\it{.79}}\,X\,A_{{\it{645}}}\\ Chl\,b \, \left( {\mathrm{\mu g/mL}} \right) = {\it{21}}{\it{.50}}\,X\,A_{{\it{645}}}-{\it{5}}{\it{.10}}\,X\,A_{{\it{665}}}\\ \mathrm{Total}\,Chl \, \left( {\mathrm{\mu g/mL}} \right) = Chl\,a + Chl\,b\\ \mathrm{Total}\,\mathrm{Carotenoids} = \frac{{1000 \times A470 - 3.27\,Chl\,a - 104\,Chl\,b}}{{229}}\end{array}$$

### Cellular oxidizing ability

The TTC (2, 3, 5-TTC) was used to assess the oxidizing ability of leaf tissue under thermal stress (TS) and control conditions^57^. 100 mg of leaf disc immersed in 50 mM phosphate buffer (pH 7.4) with 500 mg/100 mL concentration of TTC solution and incubated at room temperature. The formation of formazan in green tissues estimated at 530 nm despite 485 nm to avoid chlorophyll interference and the observations recorded as absorbance per gram of fresh weight.

### Statistical analysis

The experiments were conducted in RCBD considering three biological and technical replicates for each treatment of contrasting cultivars (HT and HS). The statistical data analysis was performed using the R Bioconductor package, GraphPad Prism 5 (San Diego, USA) and excel 2013^62^. Data considered statistically significant at a *p*-value < 0.05 using Student’s *t*-test. Data represent the mean ± SD from three independent experiments.

### RNA-seq transcriptional profiling

#### RNA extraction, cDNA library preparation, and sequencing

The third mature leaves of treated and control samples in three biological replicates from the extremely tolerant and sensitive cultivar were harvested and flash-frozen in liquid nitrogen for RNA extraction. The extraction of high-quality total RNA was performed following IRIS protocol^63^. The RNA quantification was estimated on NanoDrop 2000 (Thermo Scientific) and its quality was evaluated on both 1% formaldehyde agarose gel (MOPS) and Agilent bioanalyzer using Chip RNA 7500 series II (Agilent Technologies, USA). Total 4 µg RNA of each replicate were pooled in equimolar concentration with RIN (RNA Integrity Number) value >8 for cDNA library preparation using the Illumina Truseq^TM^ RNA Sample prep v2 LS Protocol (Illumina Inc., CA, USA). The cDNA libraries prepared were quantified on Qubit 2.0 fluorometer (Invitrogen, USA) and quality was assessed on Agilent 2100 Bioanalyzer (Agilent, USA). The paired-end (2 × 72 bp) sequencing was carried out using the Illumina Genome Analyzer IIx platform (San Diego, CA).

#### The de novo assembly, functional annotation, and differential expression analysis

The base calling and demultiplexing of raw data obtained from Illumina was performed using CASAVA *ver.* 1.8.2 pipeline. The filtering of raw reads was performed using NGS QC Toolkit considering the phred score value >30^64^. The genome-guided de novo assembly of filtered reads was performed using TRINITY RNA-Seq platform *ver*. 2.3^65^ with default parameters considering 300 bp of minimum transcript length. The assembled unigenes were further clustered based on sequence similarity (90%) using CD-HIT-EST *ver*. 4.6 clustering tool^66^. TransDecoder program was used to predict the full-length coding regions of the assembled unigenes considering the longest open reading frame and log value of likelihood function for the sequences needed (https://transdecoder.github.io/). The HMM-Scan was performed to assign protein family to the assembled unigenes, which were also annotated using NCBI’s nr, Swiss-Prot, TAIR10, EggNOG v4.5 (http://eggnogdb.embl.de/), KEGG (http://www.kegg.jp/kegg/tool/annotate_sequence.html) and plant transcription factor (http://planttfdb.cbi.pku.edu.cn/) databases considering E-value 1e−10. The Individual sample-specific reads were mapped to the de novo assembled unigenes using Bowtie2 *ver.* 2.2.4^67^ and normalized to estimate the unigene abundance. Further, the reads were normalized as TPM (transcript per million mapped reads) to estimate the unigene abundance. The unigenes abundance and their differential expression among C6017_H (HS_H), C6017_ct (HS_ct), and TV17_H (HT_H), TV17_Ct (HT_ct) were estimated using the edgeR tools^68^. The Benjamini–Hochberg FDR method was performed to adjust the *p*-values of differentially expressed unigenes for multiple testing^69^.

#### The reference-based differential expression analysis

Along with de novo analysis, the reference-based differential expression analysis was also performed using both CSS (*Camellia sinensis sinensis*) and CSA (*Camellia sinensis assamica*) draft genome^30–32^. The filtered reads of each sample were mapped to both the draft genome using tophat *ver.* 2.1.0 and assembled using Cufflink. The unigenes expression pattern was quantified using cuffquant and normalized with cuffnorm tool. Furthermore, cuffdiff was used to estimate the differential expression pattern among the samples at both gene and isoform levels. Statistically significant differentially expressed unigenes with FC > ±2 and FDR ≤ 1e−2 were extracted for the downstream analysis. The significantly differentially expressed unigenes obtained from both the de novo and reference-based differential expression analysis were further clustered based on their median expression values (FPKM), with fold change (FC) > ±2 and FDR ≤ 1e−4 in pairwise DGE. The significantly clustered unigenes subsequently used for downstream pathways and transcriptional network prediction. The identified unigenes were successfully mapped to 15 chromosomes of sequenced reference tea genome^32^.

#### The KEGG and plant reactome pathway enrichment

The significant differentially expressed unigenes in both de novo and reference-based expression profiling were subjected for GO annotation and enrichment analysis using WEGO 2.0 and AgriGO *v.*2.0^70,71^. The GO enrichment analysis was performed with plant GO slim database using Fischer exact statistical test (Hochberg-FDR adjustment cut-off <0.01). The pathway enrichment analysis was performed using KEGG and plant reactome pathway database^72,73^. The pathways annotation and curation were performed by gene set enrichment analysis using the R Bioconductor package^74^. The Fischer statistical analysis (Hochberg-FDR adjustment cut-off <0.05) was used for the identification of significant differentially enriched pathways in tolerant and sensitive cultivars to determine the heat stress-related pathways.

#### Transcriptional interactome network prediction

The pre-determined experimentally validated protein–protein interactome networks of the plant species (*Arabidopsis thaliana*, *Arabidopsis lyrata*, *Brassica rapa, Cicer arietinum, Theobroma cacao, Oryza sativa, and Zea mays*) available in string interactome database were downloaded and used for network construction^75^. The orthology of significantly DE unigenes sequences were obtained with TAIR and Swissprot protein database using blastx tool (e-value = 1e−10; % sequence identity and sequence coverage >60%). The interologous transcriptional network of heat stress-associated unigenes was created by successfully mapping the orthologs with the downloaded string interactome database and analyzed using Cytoscape ver. 3.4.0^76^. The unigenes were considered as nodes, having a significant correlation edge (FDR ≤ 0.05) with respective orthologs of the predicted network. The functional modules in the constructed network were identified using the MCODE tool which was subjected for pathway enrichment using KEGG and plant reactome database^77^. Pearson’s correlation was computed based on the differential expression profile of nodes in the predicted network to identify the co-expression between the significantly enriched pathways (*p*-value ≤ 0.05). The R Bioconductor package was used to perform pathway curation and gene set enrichment of predicted network^74^.

#### Quantitative real-time PCR (qRT-PCR) validation

Total high-quality RNA, considered for RNA-Seq analysis (extracted from third mature leaves of treated and control samples) were used for qRT-PCR validation of identified differentially expressed unigenes in this study. The first-strand cDNA was synthesized utilizing 4 µg of total RNA by Revert Aid First-strand cDNA synthesis kit (Thermo Scientific, USA). BatchPrimer3 (http://probes.pw.usda.gov/batchprimer3) was used to design the gene-specific primers of heat stress-associated unigenes (Supplementary Table S9). A reaction mixture of 20 µL reaction volume was prepared, comprising of 200 ng template cDNA, FG-POWER SYBR® Green PCR Master Mix (Applied Biosystem, USA), and gene-specific primers in StepOne™ Real-Time PCR System (Applied Biosystem, USA) instrument. The expression analysis of all the genes was performed in three biological and technical replicates each using 18 s as an internal control for normalization of expression level as used in the previous studies^4,5^. The relative expression and fold change were calculated using comparative Ct values^78^. The GraphPad Prism 5 (San Diego, USA) was used for conducting statistical data analysis, and significance (*P* < 0.05) was obtained using Student’s *t*-test^62^.

#### HSP90 inhibitor leaf disc assay and expression analysis

To assess the effect of HSP90 in HSR, a rapid bioassay with GDA (specific HSP90 inhibitor) was performed using leaf disc assay^79^. Leaf disc of 15 mm diameters was punched out from third mature leaves and floated on 5% v/v DMSO solution in water as control. For HSP inhibitor assay, 50 µM GDA solution was used and leaf disks subjected to 38 °C for 24 h heat stress mimicking similar conditions as mentioned in the Plant material section. The REL and cellular oxidizing ability (using the TTC method) were estimated from the leaf disc as mentioned in the above section (REL for membrane damage and cellular oxidizing ability). In addition, high-quality RNA was extracted from the leaf disc and processed for qRT-PCR expression profiling. The experiments were conducted in three biological and technical replicates each and statistical significance was computed using the student’s *t*-test as described in the qRT-PCR validation section.

#### Conserved motifs and structure prediction of heat shock proteins (HSP90) in tea

The complete protein domain of unigenes was extracted and the conserved motifs were predicted using MEME suite^80^. The physiological properties and cellular localization of full-length protein sequence were performed using ExPASy ProtParam^81^ and CELLO tool^82^. BLAST and HHBlits were used to perform template search against the SWISS-MODEL template library^83^. ProMod3 was used to build a model based on conserved co-ordinate between templates and targets with insertion and deletion remodeled using a fragment library, and the geometry was regularized using force field^84^. The global and per-residue model quality of the predicted structure was estimated using the QMEAN score^85^. The quality structure quality estimate score was used to estimate the accuracy of the predicted tertiary structure model using a supervised machine learning algorithm, which is calculated based on interface conservation, structural clustering, and other template features^86^. Subsequently, docking of GDA with the predicted protein structure was performed using Autodock Vina and UCSF Chimera docking tool^87,88^. Prior to docking energy minimization was performed by a universal force field using an algorithm and the best molecular structure based on binding affinity energy was visualize using Pymol ver. 2.3^89^.

## Supplementary information

Supplementary information

Supplementary Table S1

Supplementary Table S2

Supplementary Table S3

Supplementary Table S4

Supplementary table S5

Supplementary table S9
