# Supplementary material for: Underpinning the molecular programming attributing heat stress associated thermotolerance in tea (Camellia sinensis (L.) O. Kuntze)
Source: Hortic Res. 2021 May 1;8:99. doi: 10.1038/s41438-021-00532-z (PMC8087774; doi:10.1038/s41438-021-00532-z)
Supplement: Supplementary file 1 — Supplementary information [file 41438_2021_532_MOESM1_ESM.pdf]

Supplementary Information:

Underpinning the Molecular Programming Attributing Heat Stress Associated Thermotolerance in Tea (*Camellia Sinensis* (L) O. Kuntze)

Romit Seth[a], Tony Kipkoech Maritim[a,b,c], Rajni Parmar[a], Ram Kumar Sharma[a,b]*

[a] Biotechnology Department, CSIR-Institute of Himalayan Bioresource Technology (CSIR-IHBT), Palampur, Himachal Pradesh, 176061, India

[b] Academy of Scientific and Innovative Research (AcSIR), CSIR-IHBT, Palampur, Himachal Pradesh, 176061, India

[c] Tea breeding and genetic improvement division, KALRO - Tea Research Institute, Box 820, 20200, Kericho, Kenya

Running title: *Heat stress associated thermotolerance in tea*

***Correspondence:**
Dr Ram Kumar Sharma
Biotechnology Division
CSIR-Institute of Himalayan Bioresource Technology
Palampur (H.P)
India
Email: rksharma.ihbt@gmail.com
ramsharma@ihbt.res.in

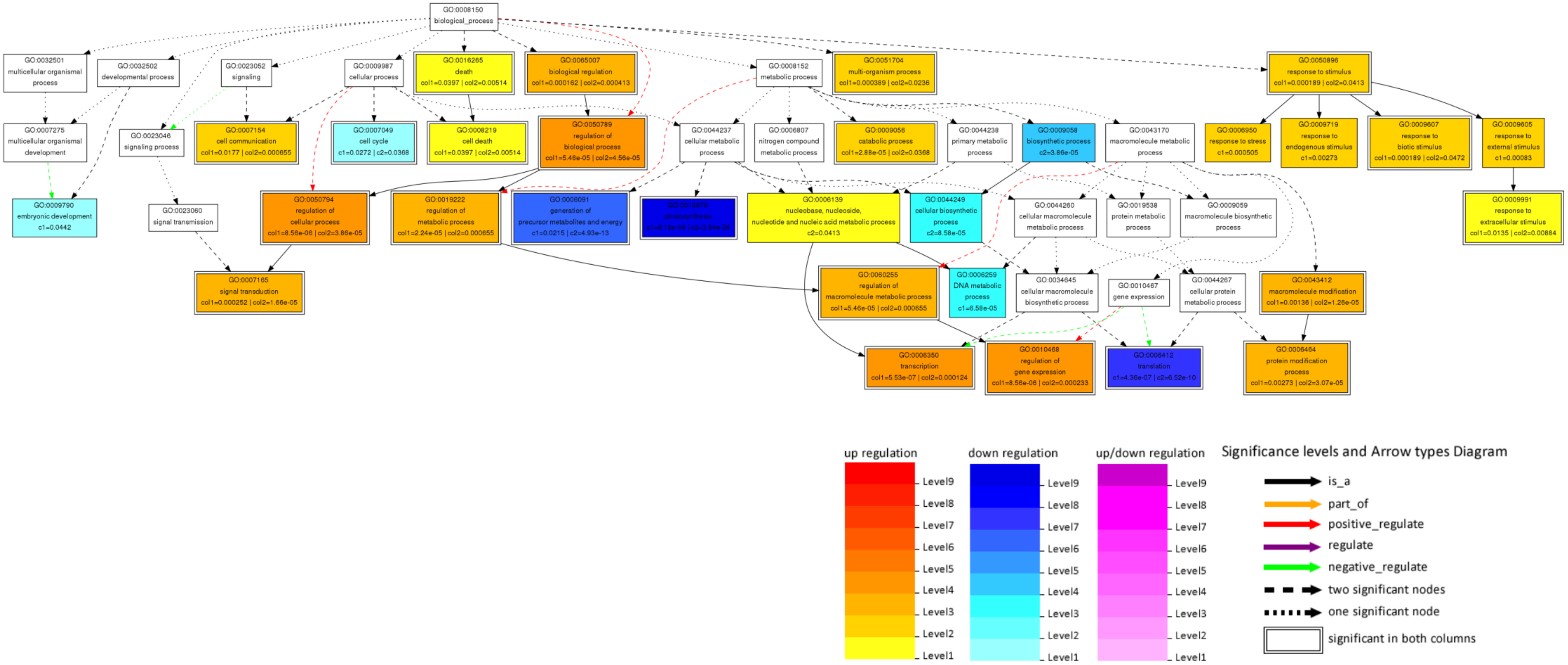

Figure S1 Differential GO enrichment of biological process in tolerant and sensitive cultivar during administration of heat stress in hierarchal clustering.

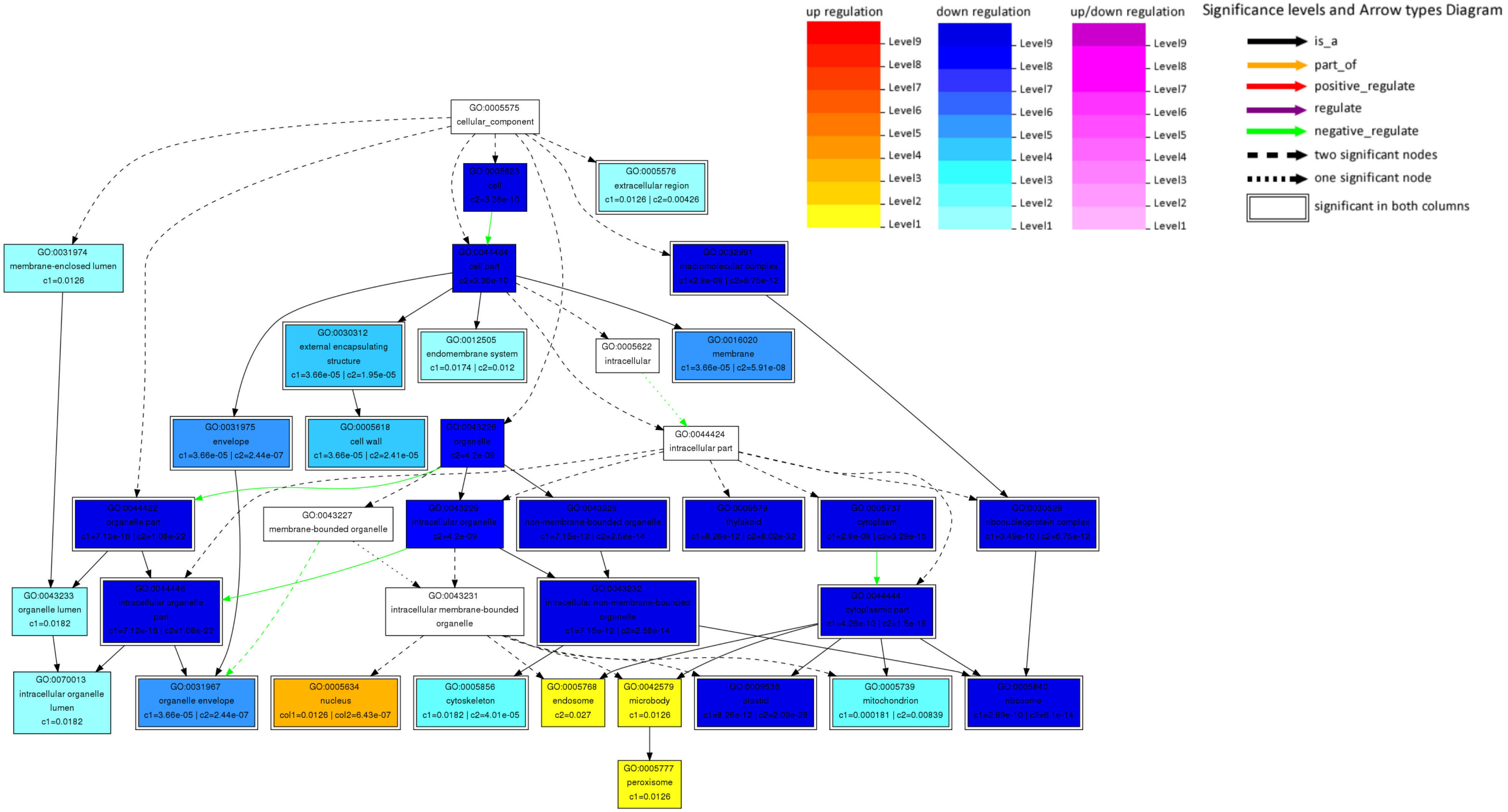

Figure S2 Differential GO enrichment of cellular component in tolerant and sensitive cultivar during administration of heat stress in hierarchal clustering.

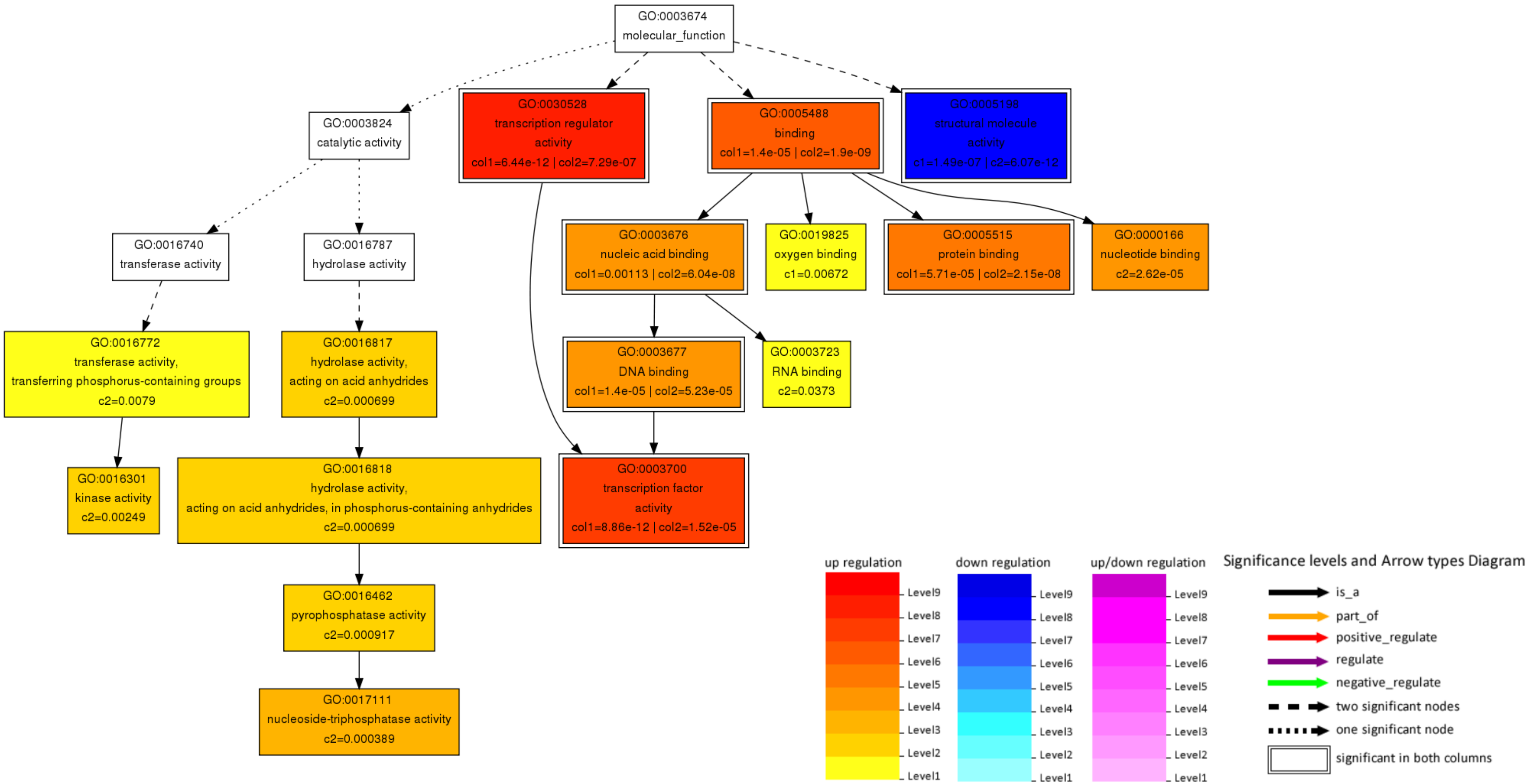

Figure S3 Differential GO enrichment of molecular functions in tolerant and sensitive cultivar during administration of heat stress in hierarchal clustering.

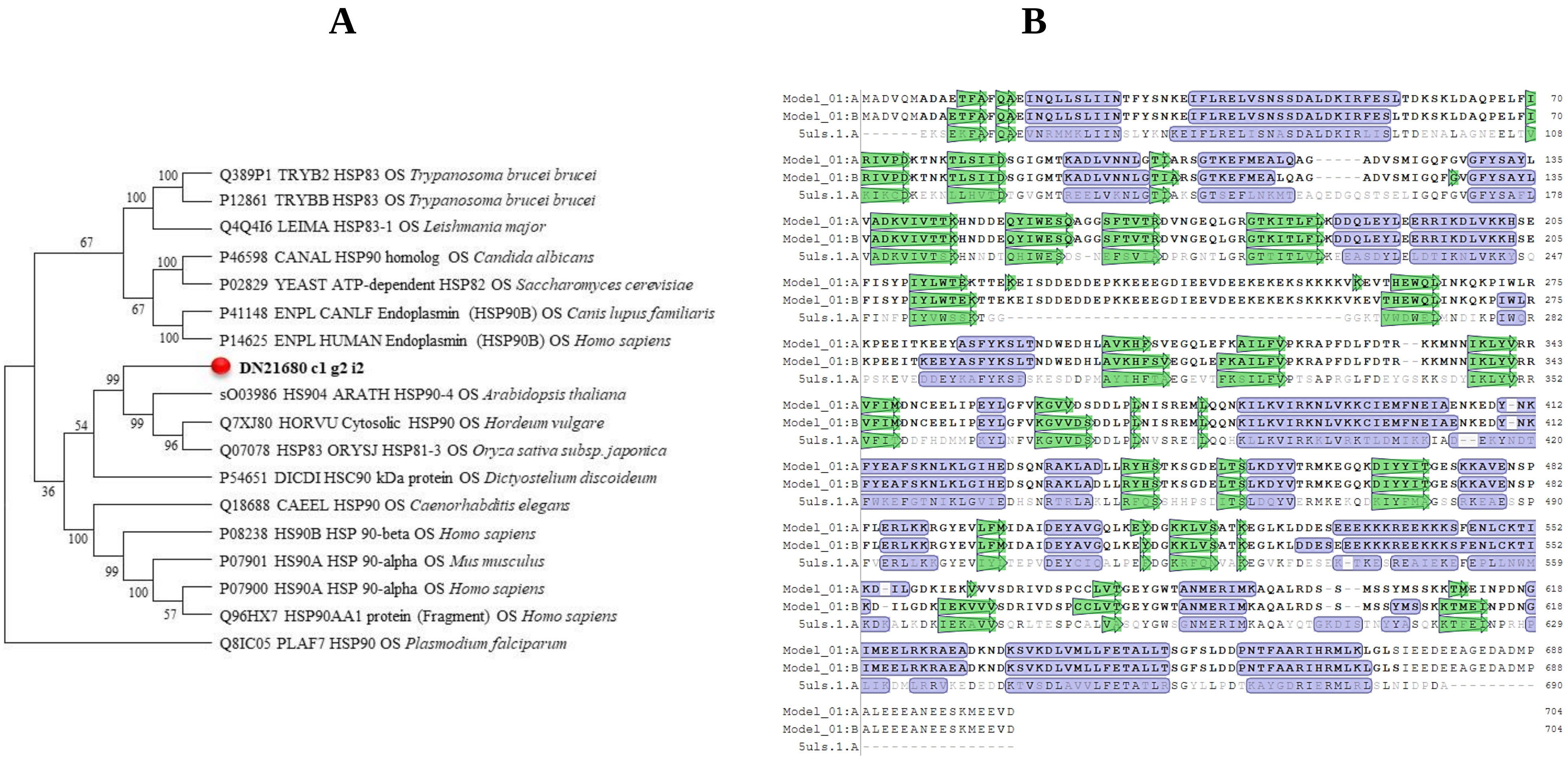

Figure S4: (A) Multiple sequence alignment of query protein (DN21680_c1_g2_i2) sequence exhibiting close structural homology with HSP90 chaperone protein. **(B)** Predicted protein homodimer sequence of DN21680_c1_g2_i2.

Supplementary Table S6: Summary of structural properties of predicted *Cs*HSP90 protein.

S. No.	***Structure properties***	
1	Predicted structure	Homo-dimer<br>(Chain A: 667 bp & Chain B: 668 bp)
2	Theoretical isoelectric point (pI)	4.99 (acidic)
3	Ramachandran plot analysis (%)	92.41%
4	Instability index (II)	42.55
5	*in vivo* half-life (λ)	30 hours (Unstable)
6	Subcellular localization	Cytoplasmic
7	Grand Average of hydropathicity (GRAVY score)	-0.633

Supplementary Table S7: List of cultivars used and their special attributes.

S. No	*Cultivar name*	*Varietal Type*	*Origin*	*Special attributes*	*Reference*
1	TV17	Assam hybrid	TRA, Tocklai, Assam, India	Drought tolerant, High Quality & High Yield	[1,2]
2	C6017	Cambod	Craigmore, The Nilgiris, UPSAI, India	Sensitivity to drought, Quality Tea Cultivar, High shoot density	[1,2]
3	HV39	China	Darjeeling, India	Moderate in quality, High shoot density	[1]
4	KANGRA-Jat	China hybrid	Tea Experiment Farm,Banuri , CSIR IHBT collection, India	Moderate to High quality, Sparsely Pubescent, High shoot density	[1]
5	Tukdah 78 (T78)	China	Tukdah Tea Estate, Darjeeling, India	Potential generative clone	[1]
6	TEENALI	Assam hybrid	Teenali, Assam, India	Moderate quality	[1]
7	TRI2024	Assam	TRI, Sri Lanka	High quality & High yield	[1]
8	TRI2025	Assam	TRI, Sri Lanka	High quality & High yield	[1]
9	TS379	Assam	TRA, Tocklai, Assam, India	High quality, Sparsely Pubescent	[1]
10	TS449	Assam hybrid	TRA, Tocklai, Assam, India	High Quality & Average Yield	[1]
11	TV18	Cambod	TRA, Tocklai, Assam, India	High Quality & High Yield	[1]
12	TV19	Cambod	TRA, Tocklai, Assam, India	High Quality & High Yield	[1]
13	TV20	Cambod	TRA, Tocklai, Assam, India	High Quality & Average Yield	[1]
14	TV22	Cambod	TRA, Tocklai, Assam, India	High Quality & Average Yield	[1]
15	TV23	Cambod	TRA, Tocklai, Assam, India	High Quality & Average Yield	[1]
16	B 6/61	China hybrid	Brookland Tea Estate, UPASI, India	Moderate quality	[3]
17	UPASI 3	Assam	Brookland Tea Estate, UPASI, India	Sensitivity to drought, High quality & High yield	[3]
18	UPASI 9	Assam	Brookland Tea Estate, UPASI, India	Drought tolerant, High quality & High yield	[3]
19	BS68	China hybrid	Tea Experiment Farm, Banuri, CSIR IHBT collection, India	High quality, Sparsely Pubescent, High shoot density	[4]
20	BGP63	China hybrid	Tea Experiment Farm, Banuri, CSIR IHBT collection, India	Sparsely Pubescent, Mid-season flush	[4]

Note: All the cultivars are grown and maintained at CSIR IHBT, Palampur, INDIA
[Latitude (32°6' 52N); Longitude (76°33' 24E); altitude of 5298 feet; mean annual rainfall 2493mm; average annual temperature 19.1°C]

[1] Sharma *et al.*, 2010
[2] Parmar *et al.*, 2019
[3] Saravanan *et al.*, 2005
[4] Unpublished

Supplementary Table S8: Five-point scale used for scoring the scorching effect of 20 tea cultivars in response to heat stress.

Score	*Degree of scorching*	*Description*
1	0-10%	Prolific flushing with no dormant shoots
2	11-25%	Wilting of leaves with few dormant shoots, few flushing shoots and some leaf fall
3	26-50%	Many dormant shoots, wilting leaves and moderate leaf fall
4	51-75%	Many dormant shoots, wilting leaves, severe leaf defoliation
5	76-100%	Severe leaf defoliation and die back, all shoots dormant and sometimes death

Supplementary Legends:

Supplementary Figures:

Figure S1 Differential GO enrichment of biological process in tolerant and sensitive cultivar during administration of heat stress in hierarchal clustering.

Figure S2 Differential GO enrichment of cellular component in tolerant and sensitive cultivar during administration of heat stress in hierarchal clustering.

Figure S3 Differential GO enrichment of molecular functions in tolerant and sensitive cultivar during administration of heat stress in hierarchal clustering.

Figure S4: (A) Multiple sequence alignment of query protein (DN21680_c1_g2_i2) sequence exhibiting close structural homology with HSP90 chaperone protein. **(B)** Predicted protein homodimer sequence of DN21680_c1_g2_i2.

Supplementary Tables:

Supplementary table S1: Functional Annotation of assembled unigenes using nr, swissprot, TAIR, pfam, interproscan, KEGG, GO and Transcription factor protein database

Supplementary Table S2: List of significantly differentially expressed 3294 key unigenes obtained by clustering based on median TPM values and pairwise DGEs [fold change (FC) > ±2 and FDR ≤ 1e-4] using de novo and both reference-based (CSA & CSS) differential expression analysis.

Supplementary Table S3: Significant Differentially enriched GO terms of (A) Biological processes, (B) Cellular components and (C) Molecular functions in heat tolerant (HT) and sensitive (HS) cultivars with respect to control.

Supplementary Table S4: Predicted transcriptional interactome network of heat stress associated pathway in tea

Supplementary table S5: Predicted transcriptional interactome network of heat stress associated pathway enriched in tolerant and sensitive cultivars of tea

Supplementary Table S6: Summary of structural properties of predicted *Cs*HSP90 protein.

Supplementary Table S7: List of cultivars used and their special attributes.

Supplementary Table S8: Five-point scale used for scoring the scorching effect of 20 tea cultivars in response to heat stress.

Supplementary table S9: List of primers used for RNA-Seq DGE validation and expresssion profiling using qRT-PCR
